# Tanzanian Nursing Students’ Experiences of Student Exchange in Sweden—A Qualitative Case Study

**DOI:** 10.1177/23779608231160923

**Published:** 2023-03-02

**Authors:** Tara Aldén-Joyce, Janet Mattson, Elina Scheers-Andersson, Paulo Kidayi, Jane Rogathi, Jenny Cadstedt, Gunilla Björling

**Affiliations:** 1 Department of Health Sciences, The Swedish Red Cross University, Huddinge, Sweden; 2 4342 Department of Health Sciences, Kristianstad University, Kristianstad, Sweden; 3 Department of Neurobiology Care Sciences and Society, 27106 Karolinska Institutet, Stockholm, Sweden; 4 Faculty of Nursing, 108094 Kilimanjaro Christian Medical University College, Moshi, Tanzania; 5 145651 Department of Nursing, 4161 Jönköping University, School of Health and Welfare, Jönköping, Sweden

**Keywords:** global nursing, nursing students, student exchange, qualitative research, nursing education

## Abstract

**Introduction:**

Needs within healthcare are changing and nurses require new skills and knowledge in global nursing. Student exchange programs in global contexts provide an opportunity to develop the necessary skills.

**Objective:**

The aim of this study was to describe Tanzanian nursing students’ experiences of student exchange in Sweden.

**Methods:**

A qualitative design was used for this empirical study. Semistructured interviews were conducted with six Tanzanian nursing students who had participated in student exchange in Sweden. The participants were recruited by purposeful sampling. Inductive reasoning and qualitative content analysis were applied.

**Results:**

Four main themes were formed; *new roles*, *experience a new culture*, *establish new competencies*, and *global work ambitions.* The findings revealed that the students experienced new approaches in Sweden, giving them new competencies and understanding. Furthermore, they increased their global perspectives on nursing and interest in working with global health issues, but they also experienced challenges in the new environment.

**Conclusion:**

The present study showed that Tanzanian nursing students benefitted from their student exchange, both personally, as well as for their future careers as nurses. More research is needed in examining nursing students from low-income countries participating in student exchange in high-income countries.

## Introduction

In 2015, the United Nations (UN) adopted an agenda for sustainable development with global goals to be met by 2030 (United Nations, 2015). They aimed to eradicate poverty, promote economic growth, and handle climate change. Health and well-being, gender equality, and quality education are fundamental parts of these goals. Global partnerships, such as nursing student exchange programs, are important for achieving these goals (Wilson et al., 2016) and for the definition of global health (Koplan et al., 2009). Nurses play an important role in the health and well-being of people all over the world (International Council of Nurses, 2020).

## Review of Literature

In today’s multicultural society, it is important that nurses do not adopt an ethnocentric view, which refers to an individual’s belief that his or her own ethnic group and culture are superior to others (Sutherland, 2002). When nurses have an ethnocentric view, this contributes to partiality to their own healthcare traditions (Almutairi et al., 2017). This in turn may result in a patient’s needs not being met thereby impacting their health and well-being negatively, leading to discrimination and negative attitudes. Nurses can gain a greater understanding of other cultures by becoming more aware of their own values (Almutairi et al., 2017). A study on Swedish nursing student exchange programs in Tanzania shows that the students experienced adversities in the situations they found themselves in (Sandin et al., 2004).

It is more common to study abroad, but still, predominantly, students from high-income countries (HICs) are visiting low-income countries (LICs). In Tanzania, university education is currently increasing, which gives more opportunities to study abroad. However, the experiences of student exchange can vary depending on the student’s country of origin and where they complete their student exchange. A review (Inyama et al., 2016) highlights the substantial research on African exchange students in general, but research specifically on African nursing students is lacking. One study states that Nigerian nursing students in the USA faced challenges such as difficulties in integration and social isolation but this lead them to develop abilities to be persistent (Sanner et al., 2002). Difficulties with integration for nursing students from Cameroon in Italy were found to be due to various reasons including language barriers and differences in culture (Masera et al., 2015). African nursing students on exchange in Australia experienced challenges with prejudice and racism. Difficulties were also reported in understanding the new culture and how to act in certain situations. However, the students stated that they had gained cultural knowledge and awareness (Inyama et al., 2015). Swedish nursing students, participating in exchange programs in various countries within and outside of Europe, became more self-aware, and made them challenge and change prior attitudes (Bohman & Borglin, 2014). A study on Norwegian nursing students in Tanzania indicated that maintaining an open attitude during exchange studies enhanced cultural awareness and competence among the students (Hovland & Johannessen, 2015).

There are several studies focusing on experiences among nursing students from HICs traveling to LICs (Bohman & Borglin, 2014; Hovland & Johannessen, 2015; Masera et al., 2015; Wilson et al., 2016), but few are investigating the reverse. By examining experiences among nursing students from LICs it can lead to a more inclusive and extensive knowledge of the subject. It is important to present a broader representation of these experiences to create a deeper understanding of other cultures and promote skills and knowledge essential to today’s nursing needs. The objective of this study was, therefore, to explore Tanzanian nursing students’ experiences of student exchange in Sweden.

## Methods

### Design

A descriptive qualitative design was used for this qualitative empirical interview study in accordance with Polit and Beck (2017). A descriptive qualitative design with semistructured interviews was used for this empirical study consisting of interviews. Inductive reasoning was applied, where the specific observations from the study lead to conclusions. A preprint of the present study has been published (Alden-Joyce et al., 2020).

### Research Questions

What are nursing students' experiences of a student exchange program?

What were their expectations of preexchange?

What were their experiences during the exchange?

What knowledge and skills have they gained?

What are the impacts postexchange?

### Sample

Purposeful sampling was used to recruit the students as this sampling method is used to find participants who can contribute the most information (Polit & Beck, 2017). A representative at the Tanzanian University was contacted who had information on which students had participated in the student exchange program at the Swedish University. Potential candidates were then sent a participant information letter with details on the aim of the study and terms and conditions. Those who agreed to participate signed a consent form. In total, six students were included in the present study.

The exchange program took place at a Swedish University in collaboration with a Tanzanian University within research methodology and thesis writing for undergraduate Bachelor of Nursing students in 2018 and 2019. The students wrote their bachelor theses during their 12-week stay in Sweden and 4 to 5 students participated in the exchange program every year. The students were part of a small project group and they either wrote one thesis together or within the same larger project. The students conducted different research topics within the area of nursing care. Teachers from both Sweden and Tanzania supervised the theses, and these teachers were part of the teacher exchange. The students took an introductory course to scientific writing and the Swedish healthcare system at the beginning of their stay.

### Inclusion Criteria

The inclusion criteria were students from Tanzanian University who had participated in student exchange programs at the Swedish University within the last two years.

### Exclusion Criteria

Students who did not want to participate in the study.

### Data Collection

Semistructured interviews were performed with six students, using a thematic semistructured interview guide. The interview guide was based on topic areas and broad open-ended questions were created to match these (Polit & Beck, 2017). The topic areas were *expectations preexchange, experiences during the exchange, knowledge, and skills gained* and *impacts postexchange*. The focus of the interviews was the participant’s experiences and the semistructured interview guide was followed. This was important to gain as rich descriptions of the student’s experiences as possible in relation to the low number of participants in the study. However, a qualitative research method aims to find detailed and varied information about the phenomena sought after rather than collecting quantitative statements about a phenomenon (Polit & Beck, 2017). In the interviews, probes were used to follow up on the answers of the participants but kept to the topic areas to capture as much relevant and rich information as possible.

All six interviews were conducted over a period of two weeks during spring 2020, and each interview lasted for an average of 45 min and was performed in English. The interviews were all done individually at a time that was convenient for the participant. The online platform Zoom was used for the interviews due to the participants being in Tanzania and the researcher in Sweden. The interviews were digitally recorded and transcribed afterward. To test the interview guide, a pilot interview was first conducted with one of the Tanzanian exchange students and lasted for 53 min. Some adjustments were made to the interview guide, and a few of the original questions were excluded as they were shown to be redundant or not relevant. After these alterations, the interview guide was then kept the same for the following interviews. The pilot interview was included in the study as the answers were conclusive to the following interviews and relevant. The saturation point in the data collection was achieved at the fifth interview. However, the sixth interview also confirmed the saturation of the collected data (Polit & Beck, 2017).

Participants received information on the study and their rights as participants prior to the interviews in the participant information letter, which was sent to them via email, this information was also repeated orally at the beginning of the interviews. The participants were also free to ask any questions regarding the study or the terms as participants before the interview commenced. Informed consent forms were collected prior to the interviews. The interviews were digitally recorded and consent to do so was received at the beginning of each interview.

Confidentiality was achieved by assigning codes to the participants; these codes were used for naming the audio recordings of the interviews as well as in the presentation of the data. To ensure confidentiality the quotations from the interviews presented in the results did not specify which students had made a particular statement, to avoid the risk of potentially identifying the participants, as the sample of participants was small.

### Institutional Review Board Approval

Ethical approval was obtained prior to the start of the study by the Research Ethics Committee at the Swedish University on 14 April 2020. This study follows the Declaration of Helsinki and the ethical guidelines in place to protect the rights and well-being of the participant (World Medical Association, 2018). The participants signed informed consent forms before the study was initiated. They were informed that their participation was voluntary, that withdrawal was possible at any time without consequences, and that data and information on the participants were to remain confidential.

### Data Analysis

The data gathered from the interviews were analyzed using qualitative content analysis. The content analysis aims to find patterns in the data and sort them into themes. The analysis was based on qualitative principles (Graneheim & Lundman, 2004). The interviews were transcribed verbatim and then read through various times to become familiar with them. The aim of the study guided the analysis and meaning units, sentences that represented concepts related to the aim of the study, were then chosen from the text. The meaning units were condensed while still conserving their meaning. The condensed meaning units were coded and grouped into subthemes. During this process, the researchers discussed the naming and meaning of each subtheme. The meaning units, condensed meaning units, codes, and subthemes were classified as manifest content and descriptions as seen in the data. In the last part of the analysis, themes were created and discussed thoroughly by all the researchers, representing the underlying meaning, latent content. These themes represent the result of a systematic and rigorous analysis process validated by researchers who are experts in the field.

Examples of the data analysis are presented in Table 1.

**Table 1.** Examples of Meaning Units, Condensed Meaning Units, Codes, Subthemes, and Themes.

Meaning unit	Condensed meaning unit	Code	Subtheme	Theme
“But also, another thing the difference of interaction, between the teacher and the students. In Sweden it was very good that at any time when you have a problem you can try to contact the teacher at any time and she, he or she, will be able to give you the feedback in advance. But here we are trained in the sense of, the sense of, there is a very big gap between teacher and students.”	The interaction between teachers and students in Sweden was good, you can contact the teacher at any time if you have a problem. Here there is a very big gap between teachers and students.	The interaction and relationship between teacher and students were good and more equal	Education system	New roles
“But that challenge also extended to the time that I was going to, we were going to find our needs, like foods in the supermarkets and everything. So, because everything is in Swedish, so you will have to ask what is, what this is or you have to look the picture to see exactly what you want because sometimes you do not get it really because it's in Swedish. So, language was another big problem.”	Language was a problem in the supermarkets when everything was in Swedish and we did not understand it	Encountering language barriers	Challenges in a new culture	Experience a new culture

## Results

The results in this study describe the experiences of Tanzanian nursing students on student exchange in Sweden.

### Sample Characteristics

In total, six students, who had participated in the exchange program in Sweden in 2018 and 2019, were included. All participants were in their third year of their four-year Bachelor of Nursing program during the time of their exchange. Three of the participants had taken part in the exchange program in 2018 and three in 2019. The students who had participated in 2018, had since then graduated and two of them were performing their one-year postgraduate internships as nurses. The third student had taken a job project before starting the internship. The students from the 2019 exchange program were in their fourth and final year of nursing studies.

The demographic data of the informants is presented below in Table 2.

**Table 2.** Demographic Data of the Participants.

Participants	Age	Gender	Year of exchange programme	Course completed	Duration of the exchange
Student A	23 years	Female	2019	Bachelor thesis	12 weeks
Student B	27 years	Male	2019	Bachelor thesis	12 weeks
Student C	25 years	Male	2018	Bachelor thesis	12 weeks
Student D	23 years	Female	2019	Bachelor thesis	12 weeks
Student E	23 years	Female	2018	Bachelor thesis	12 weeks
Student F	25 years	Male	2018	Bachelor thesis	12 weeks

### Research Questions Results

The results from the research questions emerged and four main themes were formed; *new roles*, *experience a new culture*, *establish new competencies*, and *global work ambitions.* Nine subthemes were created with the themes, and the themes and subthemes are presented in Table 3.

**Table 3.** Themes and Subthemes.

Themes	Subthemes
New roles	Education system
	Nursing role
	Gender roles
Experience a new culture	Challenges in a new culture
	Support from student mentors
Establish new competences	Interaction skills
	Confidence and discovering potential
Global work ambitions	International opportunities
	Working with global health issues

### New Roles

The student exchange in Sweden exposed Tanzanian nursing students to different approaches in various areas, these areas are described in the three subthemes: *education system, nursing role*, and *gender roles.*

#### Education System

The students found differences in the education system in Sweden compared to Tanzania, first, the research method that they used for their theses was new to them. Most of the students conducted qualitative interviews for their bachelor theses, something that they had not done earlier. They explained that in Tanzania the nursing undergraduates usually did quantitative research and they had little previous knowledge about qualitative research. They described that this new knowledge had led them to have a broader understanding of different research methods. This new knowledge also opened possibilities of conducting further qualitative research and many of the students expressed increased interest in pursuing more research in the future.

So, I have gained access to knowledge about that. And I think it’s, it will help me even if now someone tell me can you help me to conduct qualitative research? I’ll be able to do that.

The students pointed out the difference in the way the schedule and classes were organized in Sweden compared to Tanzania. They expressed these new approaches as positive; that classes started on time and that when cancellations were made that the students were notified. They also described that the time in class was limited, and the students had a lot of time for self-studies. A contributing factor to this was that many exercises and materials could be accessed online, and the students could therefore choose to study for example from home or from the library.

I mean that students have a lot of time for self-study, they do not spend a lot of time in class. They are exposed to libraries and online learning and other things.

A benefit of self-studies and not having to spend all day in class was explained by one student as enabling time to deepen knowledge and not just learn from what the teacher says. Furthermore, the student expressed that having seminars focusing on discussions put higher requirements on the students to have knowledge than just listening to the lecturer. The access to spaces for studying like the library and group rooms as well as good Internet and printing facilities were described as assisting learning.

Another aspect that the students emphasized was the good relationship and interaction between teachers or supervisors and students. They expressed that the relationship was more equal to what they experienced in Tanzania. This permitted the students to advocate for their ideas while accepting suggestions from their teachers. The teachers were not seen as superior, and the students considered them as their friends.

But also, another thing the difference of interaction, between the teacher and the students. In Sweden it was very good that, at any time when you have a problem, you can try to contact the teacher at any time and she, he, or she, will be able to give you the feedback in advance. But here we are trained in the sense of, the sense of, there is a very big gap between teacher and students.

The excerpt above shows how the relationship between teacher and student was based on collaboration, and open discussions could be held where they could negotiate rather than the teacher always believing that they were right.

#### Nursing Role

Although the students did not participate in clinical practice, many of them interviewed nurses for their theses and in this way, they were presented with the work and role of nurses. They discovered that nurses in Sweden have an independent role and that each healthcare profession is respected for their competence. They also learned that working with an interprofessional approach was essential for the benefit of the patient, as well as learning to work in a team. All the nursing students confirmed the importance of the independent nursing role which they expressed as lacking in Tanzania, where doctors often were seen as superior and would not respect the specific knowledge of the nurse. They also talked about how nurses provided holistic care to patients in Sweden.

Yeah, I think some things I will…I will…I will use some things which I gain in Sweden, I will use in Tanzania also like the way how that the nurses are independent in their decision. And also, the way how they…they got…they provided the, the holistic care, you know in Sweden they provide the holistic care.

The students expressed that they would like to implement more holistic care in Tanzania, but they also stated that a problem in Tanzania was that a nurse took care of many patients, sometimes up to 30, and therefore it was hard for them to get the time to provide holistic care.

#### Gender Roles

The students were exposed to the gender roles in Swedish society and also to specific topics that some of them covered in their theses. They all declared that the status of women was higher, and that gender equality was better in Sweden.

And also, another difference it is about empowerment and in Sweden women are so empowered compared to Tanzania.

They expressed that women were free to study and work with what they wished. A contributing factor that was mentioned was the parental leave in Sweden where men also stay at home with the children, and this promoted gender equality. One student pursued a thesis about sexual harassment among nursing students and learned about the laws in place to protect women and expressed wanting to improve these rights for women in Tanzania.

### Experience a New Culture

The nursing exchange students had countless new experiences in Sweden and although they described their experiences as mainly positive, they also faced challenges in everyday life and matters related to their studies. It was the first time abroad for all of them and they encountered many differences in the new culture. This theme is divided into the following subthemes, *challenges in a new culture* and *support from student mentors*.

#### Challenges in a New Culture

One of the most noticeable aspects of the new culture that the students talked about was the difficulty in interacting with Swedish people. They noted that Swedish people are not so sociable with people they do not know and rarely start conversations beyond greetings. They stated that it was hard to start conversations with people for example on public transport, which they pointed out as a big cultural difference compared to Tanzania, where they were used to having conversations with strangers on buses. That Swedish people were often busy and in a hurry was also a reason given for them being not so sociable.

In Sweden, the people they do not talk to strangers. That is the difference, but then we asked our mentors say that’s the culture, everyone is just busy, trying to be conscious with timings. Most people are shy and ok, just get it. I was like, this is a new culture, and I should not interfere with it.

However, the students recounted that it was easier to interact and get to know people in Sweden in specific settings and with people with who they had things in common, for example, other students or when attending church. Their student mentors were mentioned as a good way to meet people, as they were introduced to their friends and families.

Another challenge that the students encountered was language barriers in some situations, although they specified that many people in Sweden could speak English and they often found people that they could communicate. They described that it was mainly in supermarkets and when using the transportation system that they encountered language barriers. They often found someone who could help them, or they would use their phones to translate information.

Ah, as I have said earlier, Swedish people, they are very kind and humble so whenever we find a communication barrier, we tried to reach out to potential people whom we saw.

An additional cultural difference that was described as a challenge was time management in Sweden; things were done strictly on time and you should be time conscious. They related that the public transport was very punctual, coming to class on time, and respecting deadlines. After adapting to this new attitude to time, the students perceived being on time as a good thing. They had greater respect for the time after returning to Tanzania and many expressed that they now, sometimes, become frustrated with the relaxed attitude to time in Tanzania. The students said that being punctual during their exchange had influenced them and made them more aware of time.

Okay, it has…it has actually influenced me so much and I have made a lot of positive changes into my life as a nurse, but also as a person, because I now make a, the on-time appointment, ha, ha.

Other challenges that were mentioned were the use of new technology such as paying with a card instead of cash and using cards in the transportation system. It took them some time to get accustomed to the transportation system as it operated very differently from Tanzania. The cold weather was also brought up as a challenge for them but they got used to it after a while.

So those are the challenges, weather, life system. And another challenge that I can say it was the language, language barrier it was challenge but we used the method which also was potential for us to, to get solutions and we coped with everything. Yeah, the other, those challenges but we're able to manage them and we learnt a lot from them yeah.

All the students concluded that they learned a lot from the challenges that they faced during the exchange and that it enabled them to develop coping skills. Being exposed to a new environment and a new culture increased their confidence in knowing that they were able to manage in the future when confronting such challenges.

#### Support From Student Mentors

The Tanzanian nursing students were each appointed a student mentor in Sweden, these mentors were nursing students at a Swedish university. The student mentors and the Tanzanian students had email contact before the exchange and the student mentors answered questions that they had about Sweden. During the exchange, the mentors were available to help with whatever was needed, and receiving this help and support was expressed by all the students as extremely beneficial to them.

Yeah really, very, it helped us a lot of cause, you know mentors help us to, to get acquainted with the environment. Sweden was a, particularly Stockholm it was the new, new image to us. We have never been there before, so having mentors they helped us to at least be conversant with the transport in system, basics in cultural things some etiquette in, in transportation areas or in using escalators like that.

The student mentors were able to provide help in all areas ranging from practical issues to cultural concerns and showed the Tanzanian students the way to the school and how to use the transportation system. One student related an incident when they had trouble using their laundry machine and called a mentor who came to their house to help. The student mentors were also important for the social life of the Tanzanian students; they visited places in Stockholm and arranged dinners together.

### Establish New Competences

The students stated that they gained and learned a lot from their exchange through the new environment that they were exposed to. They also developed on a personal level and the subthemes related to this theme are *interaction skills* and *confidence and discovering potential*.

#### Interaction Skills

During their stay in Sweden, the students got the chance to interact with people from different backgrounds. The students described that interacting with new people from a different culture was a challenge at first but that resulted in enhanced skills in interaction. This provided the students with a broadened understanding of other perspectives and aided communication.

But also, I can easily communicate with the friends from abroad because I know how they behave mostly and how they perceive stuffs.

They made friends with people who had different mindsets and it influenced how they perceived others. They stated that it had made them more aware and tolerant and that it was now easier to interact with different people after returning to Tanzania. They concluded that this new ability and knowledge would help them in their work as nurses to communicate with patients from different backgrounds.

#### Confidence and Discovering Potential

The students expressed that the exchange had increased their confidence, which benefitted them personally, but also for their future careers as nurses. Some of the students also discovered potentials that they were not aware of before the exchange. Discovering potential also influenced their futures and the student cited below proceeded to write a book about nursing after discovering skills in writing during the exchange program.

I can simply say that myself and my exploration that I had in Sweden has greatly opened a lot of opportunities but also has restored me with such a confidence I never had before. Because I, since came from there, I recognised some potentials, whom I did not know that I had before. For example, I did not know if I was having such a good writing skill.

Many of the students explained that defending their theses in front of other people had increased their overall confidence as well as being more comfortable speaking in public. One student described that he now felt comfortable speaking in mass or other gatherings.

### Global Work Ambitions

The exchange program influenced the student's aspirations for their future careers as nurses as many of them saw possibilities that they had not been aware of before the exchange. They also expressed a passion for working with global health issues. This theme comprises two subthemes: *international opportunities* and *working with global health issues*.

#### International Opportunities

Many of the students expressed that the exchange program had changed their mindset about what they could work with and achieve as nurses, before participating in the exchange program they were not aware of the potential of working internationally as a nurse. When they chose to study nursing, they had not considered the possibility of studying or working abroad but only thought about working in their own country.

I, before I went, I never realised that you could go work with anybody and I never realised that nursing was a global career. I thought that by studying to become a nurse maybe I can work in my country or in the place where I will be selected or to join with the government.

All students expressed interest in working internationally and that the exchange had given them the skills to do so. The student exchange facilitated them to develop abilities to adjust and cope with a new environment and they were therefore confident to work abroad in the future. They stated that working internationally and being exposed to other people’s cultures would enable them to give better care to their patients.

…if I work internationally, I still, I will get a lot of experience and so I will be able to use my skills that I got as an exchange student to work for other people. But also, I will be able to explore other people's cultures and use them and use the experience to care for the patients.

One student explained that after using online resources in Sweden he learned how to find online opportunities for work and study. All the students said that they were inspired by the exchange to do further studies and research in the future.

#### Working With Global Health Issues

The students developed increased awareness and knowledge about global health and specifically the UN Sustainable Development Goals (SDGs; United Nations, 2015), which were part of the curriculum. They expressed increased passion and interest in working with global health issues, especially in improving the health status of Tanzania.

So, I mean that is one among the Sustainable Development, Development Goals to decrease, to improve the health status of the patients. So, I really, really like to improve but the health status of our people. But especially in the case of the mothers and child.

## Discussion

The findings revealed that the students experienced many new situations in Sweden and gained significantly from the exchange program, both personally, as well as for their future careers as nurses.

### Benefits of Nursing Student Exchange

Studies show that nursing students in exchange programs reflect on practices and aspire to improve procedures in their own countries (Green et al., 2008; Morgan, 2019; Thompson et al., 2000). The students in the present study were inspired by the new approaches that they encountered in Sweden. In the Swedish education system, they encountered new research methods, more self-study time, and online resources as well as a more equal relationship between teachers and students. They expressed that they had gained new skills and an increased understanding of different approaches that could benefit them in their future work as nurses. The students were exposed to the nursing role and gender roles in Sweden through their theses as they interviewed nurses. They were influenced by the independent nursing role and holistic care provided in Sweden and hoped to implement these practices in Tanzania. Similar findings were shown in a study (Maltby et al., 2016), where the students reflected on the status of women and the gender roles that they encountered in Sweden. They found that female nurses were more empowered in Sweden compared to Tanzania, something that they wanted to address in their country.

While many studies on nursing students examine the experience of clinical practice in another country, in the present study, the students only participated in theoretical courses, but gained similar professional and personal skills as those doing clinical practice reported in other studies (Gosse & Katic-Duffy, 2020; Nielsen et al., 2020; Thompson et al., 2000; Tommasini et al., 2017). The students in the present study benefitted from the student exchange by becoming strengthened in their roles as future nurses, which is consistent with previous research (Gosse & Katic-Duffy, 2020; Nielsen et al., 2020). They became motivated and confident that they could work in any area that they wished and became aware of new possibilities for work and further studies that they had not considered before participating in the exchange program (Nielsen et al., 2020). In our study, the students developed interaction skills and cultural competence as emphasized in many other studies as a benefit of nursing student exchange (De Natale & Waltz, 2015; Edmonds, 2012; Hovland & Johannessen, 2015; Maltby et al., 2016; Thompson et al., 2000). Being exposed to a new environment and culture gave the students a wider perspective on people from different backgrounds and how to communicate with them in caring situations, something that will empower them as nurses when taking care of patients.

The students also gained personally from the exchange. Defending their theses in front of many people increased their confidence and ability to speak publicly and argue for their position. This is a common feature among nursing students, where experiencing a new culture and leaving their comfort zone increases confidence (Bohman & Borglin, 2014). Additionally, nursing exchange studies can promote empowerment and awareness of own potential (Mattila et al., 2010), which was true for some of the Tanzanian nursing students in the present study. One student discovered skills in writing during the exchange and proceeded to write a book on nursing after the exchange.

### Challenges in Nursing Student Exchange

Although the students described their experience as primarily positive, they also faced challenges in the new environment and new culture. Encountering language barriers has been addressed by numerous studies on a nursing student exchange (Gower et al., 2017; Maltby et al., 2016; Masera et al., 2015; Mattila et al., 2010; Sanner et al., 2002), and is a challenge that the Tanzania nursing students were confronted with. Even though they were able to communicate well with many people in their surroundings in English, such as their supervisors and student mentors, they encountered language barriers for example when going to the supermarket and using public transport.

Another challenge that the students faced was difficulties in getting to know new people apart from their supervisors and student mentors. This is a common challenge for nursing students on exchange programs where integration has been difficult and low interest from locals to interact has been reported (Masera et al., 2015; Mattila et al., 2010; Sanner et al., 2002). In the present study, the students described this as a cultural issue, rather than low interest from others to get to know them. They expressed that in Swedish culture it is difficult to approach someone you do not know and start a conversation, and they recognized that it was easier to get to know new people in certain settings where they met people with common interests. They socialized a lot with their student mentors; being introduced to new people through them. Implementing adequate support systems to increase the integration and benefits of nursing student exchange programs is recommended but reported as often lacking and something to be improved (Gower et al., 2017; Sanner et al., 2002).

### Global Perspectives

In this study, the students did not show any signs of ethnocentrism, as they were open to the new culture and the differences they encountered. The cultural difference and challenges regarding interaction in Sweden were not expressed by the Tanzanian students in negative terms as they recognized and accepted this difference (Masera et al., 2015). The fact that students experience ethnocentrism (Edmonds, 2012) could not be corroborated in this study. This implies that, whether students develop ethnocentrism or not, may be highly context-dependent.

Many studies on nursing exchange programs present students from HICs being exposed to poverty for the first time in LICs (Gower et al., 2017; Hovland & Johannessen, 2015; Sandin et al., 2004). The importance of providing care and advocating for these vulnerable groups was acknowledged. They increased their understanding of global health issues and the SDGs (United Nations, 2015) and interventions to deal with these. The students also wished to work internationally and were more confident to seek study or work opportunities abroad in the future as they now had skills to cope with a new environment.

## Strengths and Limitations

Purposeful sampling was convenient for this study but can also lead to less variation regarding participants and collected data (Polit & Beck, 2017). Data analysis used for this study was content analysis and actions were taken to ensure trustworthiness (Graneheim & Lundman, 2004). Credibility was attained by providing truthful data, this study conducted semistructured interviews that encouraged the participants to talk freely while exploring topic areas (Polit & Beck, 2017). All the collected data were included in the analysis, examples of the analysis process were presented, and quotations from the transcripts were. The participants consisted of an equal amount of men and women and the researcher and participants did not have any prior relationship. Transferability relates to the possibility of the findings being applied in another setting or group (Polit & Beck, 2017). Although the sample size was small valuable and consistent findings were gathered. However, a study at a later stage including more students may give more variation to the data.

## Implications for Practice

The findings can be used to understand the benefits and challenges of student exchange programs to develop them in relation to specific goals in the curriculum. The findings also indicate the importance of support during the student exchange program, such as student mentors, to promote integration, and extracurricular activities, and enable a positive experience. It can be recommended that other nursing student exchange programs implement similar practices for support. This study suggests that nursing exchange programs increase knowledge of global health and global nursing. The nursing curriculum should also include global health to prepare nurses for the needs of the global community and a sustainable future.

## Conclusions

This study showed that Tanzanian nursing students benefitted from their student exchange, both personally, as well as for their future careers as nurses. The findings support earlier research that nursing exchange programs and partnerships promote knowledge in global health and global nursing. The exchange program enabled Tanzanian nursing students to develop essential skills in global nursing such as cultural competence, holistic care, and advocacy. More research is needed to improve and promote the benefits of these programs and research examining the specific experiences of nursing students traveling from LICs to HICs.
